# Research on the Evolution of “Ren” and “Li” in *SikuQuanshu* Confucian Classics

**DOI:** 10.3389/fpsyg.2021.603344

**Published:** 2021-04-16

**Authors:** Bo Hu, Fugui Xing, Miaorong Fan, Tingshao Zhu

**Affiliations:** ^1^Institute of Psychology, Chinese Academy of Sciences, Beijing, China; ^2^Department of Psychology, University of Chinese Academy of Sciences, Beijing, China

**Keywords:** big data, culture, Confucian, Ren (humaneness or benevolence), Li

## Abstract

Confucian culture has always been the most glorious component of Chinese culture. Governing the mainstream world of China for more than two millennia, it has cast a profound and long-lasting influence on the way of thinking and cultural-psychological formation of the Chinese people. Confucianism emphasizes caring about others with benevolence and governing a state with ethics, reflecting the importance of moral principles for politics. “Ren” and “Li” are important parts of the core values of Confucianism, so analyzing the differences between them and their evolution is of great significance for further understanding Confucian culture. This paper selected 132 classic Confucian works from *SikuQuanshu*, a large collection of books compiled during the Qianlong’s reign of the Qing Dynasty (1636–1912), to calculate the use of frequency of “Ren” and “Li” in those books by means of big data. Then the data was analyzed to show the development trajectory of “Ren” and “Li” from the Spring and Autumn period (770–476 BC) to the Qing Dynasty, providing a new perspective for the study of Confucian culture. The analysis result shows that from the Spring and Autumn period to the Qing Dynasty, both the frequencies of “Ren” and “Li” record a peak and a bottom: “Ren” has its peak in the Sui and Tang period (581–907) while “Li” reaches its climax in the Wei and Jin period (220–420); both “Ren” and “Li” hit their bottom during the Yuan Dynasty (1279–1368). The average frequency of “Li” is higher than that of “Ren” during most of the time (eight dynasties and periods). In general, “Li” is more frequently referred to in classic Confucian works than “Ren,” especially in those of the pre-Sui and Tang era. The An-Shi Disturbances in the Tang Dynasty may mark an important turning point for the frequencies of “Ren” and “Li” in classic Confucian works.

## Introduction

The Chinese culture traces back to the Spring and Autumn period when the Hundred Schools of Thought flourished. Among the different schools of thought, Confucianism enjoys the most glory. Since founded by Confucius, Confucianism gradually became the official system of thought and behavior during the ancient period of China, especially after the ideological development made by Zhongshu Dong in the Han Dynasty (BC 206–220). Dominating the Chinese society for over two millennia, Confucianism has produced far-reaching impacts on the way of thinking and cultural-psychological formation of the Chinese people (Li, 1980, p. 6).

Confucianism emphasizes caring others with benevolence and governing a state with ethics, reflecting the importance of moral principles for politics. It builds a moral framework consisting of three cardinal guides, five constant virtues, four cardinal principles, eight virtues, etc. Among them, “Ren” and “Li,” two components of the five constant virtues are the core of pre-Qin Confucianism and important parts of traditional Chinese moral values (Zhao, 2011, p. 1). “Ren” and “Li” have complicated meanings and share a complex mutual relationship; to put it simply, “Ren” refers to inner moral sentiments while “Li” is extrinsic behavioral norms (Wang, 2018, p. 2). Compared with other morals and principles, “Ren” and “Li” boast a longer history and have been constantly interpreted throughout the development of Confucian culture. Therefore, analyzing the differences between them and their evolution is of great importance for further understanding Confucian culture.

Nearly all previous studies of “Ren” and “Li” adopt qualitative research methods, with the focus on their meanings, mutual relationship, social functions and the comparison of their importance and so on. It is generally considered that “Ren” is the ideological core of Confucianism (Zhong, 2016, p. 1), representing ideologies and moral concepts (Zhao, 2011, p. 4), whereas “Li” is the outward manifestation of “Ren,” representing institutions and behavioral norms (Yang, 2017, p. 2); “Ren” and “Li” share a complementary relationship like a pair of wings for a bird and two sides of wheels for a car (Wang, 2018, p. 2). However, longitudinal quantitative analysis over a long span of time has not been employed in Confucianism studies by far. Compared with qualitative analysis, quantitative analysis is able to analyze massive classical Chinese materials in a relatively short time; it has greater advantages in displaying a variable’s changes of different degrees over time and the patterns of such changes, thereby promoting the studies of Confucianism to go further and deeper.

In recent years, cultural product analysis has been increasingly preferred in examining cultural changes, and an archive of written language is suitable for cultural product analysis (Hamamura and Xu, 2015). Rong Zeng et al. used the Google Ngram Viewer to calculate changing frequencies of certain key words to study the implications of social and political changes on cultural values in China over the last 40 years (Zeng and Greenfield, 2015). Wu et al. (2019) used similar approaches to analyze cross-cultural differences in affectionate communication. As books are public tangible representations of culture, the analysis of the use frequency of key words in books over time can be a relatively objective and effective approach for cultural evolution studies.

This paper bases its research on the big data of classical Chinese extracted from *SikuQuanshu* (the Complete Imperial Collection), with a focus on the Confucian classics (“classics” herein refer to officially recognized theoretical works or widely circulated works), and then obtained electronic versions of the Confucian classics included in *SikuQuanshu* from reliable sources to track the changes in the frequencies of “Ren” and “Li” in those books over a long period of time. The tool used to extract keywords is the Classical Chinese Linguistic Inquiry and Word Count (known as CC-LIWC) which is constructed by Computational Cyber-Psychology Lab, Institute of Psychology, Chinese Academy of Science (Fan, 2020). Finally, we examined the differences in average frequencies of the two keywords to study which one of them is more frequently discussed in those Confucian classics.

Given the role “Li” plays in safeguarding political and social order, we think that the ancient ruling class may have laid more emphasis on “Li” than “Ren.” Therefore, this paper hypothesizes that: “Li” is more frequently discussed in Confucian classics than “Ren.”

## Materials and Methods

### Data Selection

*SikuQuanshu*, compiled during the Qianlong’s reign of the Qing Dynasty (1636–1912), is a complete collection of books in the four branches of literature, and includes many Confucian classics (Wang, 2017, p. 10). These books are categorized into Jing, Shi, Zi, and Ji, and the majority of books under the category of Zi are theoretical works of master philosophers. Under the first subcategory of Zi are Confucian Books, representing the essence of Confucianism of all times (Wang, 2017, p. 21). This paper follows two principles when selecting Confucian books: “classics first^1^” and “quality before quantity.” In the process of compiling *SikuQuanshu*, Confucian works were further divided into Zhulu and Cunmu. Books falling into the type of Zhulu were confirmed to be well verified and highly credible while those classified into Cunmu were considered as inferior ones full of faults and forges or non-verified (Wang, 2019, p. 1–7). This paper bases its research only on books classified into Zhulu after cautious deliberation.

It should be noted that some scholars believed books included under the section of Confucian Books in *SikuQuanshu* were incomplete (Wang, 2017, p. 10), which is not completely groundless. We found three major issues in this regard: (1) Some Confucian classics were not included under the section of Confucian Books, such as *Thirteen Classics* and *Four Books*; (2) classics by Zhongshu Dong, a representative Confucian figure in the Han Dynasty (BC 206–220), were not included under the section of Confucian Books; (3) classics of Jiuyuan Lu, Founder of Philosophy of the Mind (a school of Neo-Confucianism), and Yangming Wang, a leading figure of the Philosophy of the Mind, were also not included under the section of Confucian Books. Instead, the above books were mainly included under the categories of Jing and Ji in *SikuQuanshu*, which resulted from the classification standards of *SikuQuanshu* on the one hand and the Philosophy of the Mind being rejected by the official at that time on the other hand (Wang, 2017, p. 32). Nevertheless, it is generally recognized that *Thirteen Classics* and *Four Books* are classic Confucian works and that Zhongshu Dong, Jiuyuan Lu, and Yangming Wang are prominent figures of Confucianism. We think that Confucianism research without these books will lead to biased analysis results, so our research also covers *Thirteen Classics* and *Four Books* as well as classic works by Zhongshu Dong, Jiuyuan Lu, and Yangming Wang.

To sum up, our research selected 132 Confucian books in total containing some 16 million Chinese characters, including 113 books falling into the section of Confucian Books in *SikuQuanshu*, 15 works from *Thirteen Classics* and *Four Books* (since both include *Analects of Confucius* and *Mencius*, we only selected *Great Learning* and *Doctrine of the Mean* from *Four Books*), two works of ZhongshuDong (*Ju XianliangDuice* and *Chunqiu Fanlu*, also known as “Luxuriant Gems of the Spring and Autumn”), one work of Jiuyuan Lu (*Xiangshan Ji*), and one work of Yangming Wang (*Wang WenchengQuanshu*). We accessed these books in txt format from such e-book platforms as www.guoxuedashi.com and then proofread these electronic versions according to the *SikuQuanshu* published by Taiwan Commercial Press, which is a photo copy of the version stored in the Wenyuan Library (visit skqs.guoxuedashi.com for detail), to ensure their liability.

### Data Analysis

As shown in the Supplementary Material, this paper lists the 132 books by title, author and publication dynasty (nine dynasties and periods involved); the start year and the end year of each dynasty or period are specified respectively; the average of the publication time of each book is calculated as x-axis values on the line charts.

First, we used CC-LIWC to calculate the number of occurrences of “Ren” and “Li” in each book and the total number of words of each book. We also calculated the number of Confucian works and the numbers of occurrences of “Ren” and “Li” by dynasty or period (see Table 1).

**Table 1:** The number of Confucian books by dynasty or period and the numbers of occurrences of “Ren” and “Li” in them.

Dynasty or Period	The number of Confucian books	The number of “Ren”	The number of “Li”
Spring and Autumn (770–476 BC)	6	166	831
Warring States (475–221 BC)	8	397	1198
Qin and Han (BC 221–220)	14	1047	2195
Wei and Jin (220–420)	2	150	444
Sui and Tang (581–907)	5	241	325
Song (960–1279)	45	16204	16630
Yuan (1279–1368)	4	108	265
Ming (1368–1636)	27	7559	10408
Qing (1636–1912)	21	4596	7369

**Figure 1:**
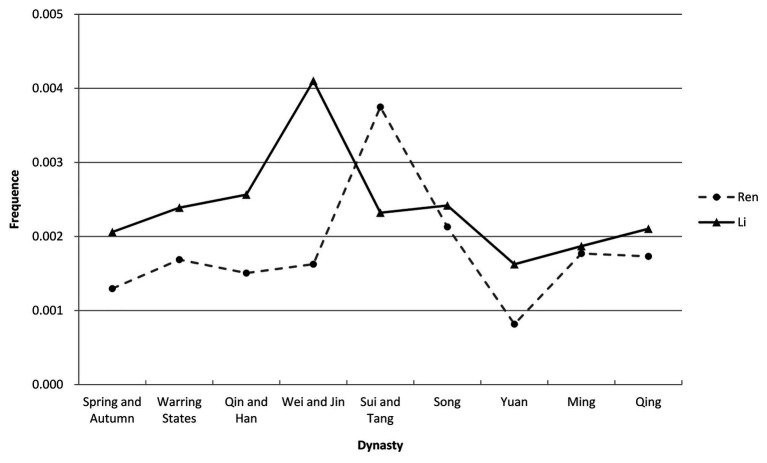
The evolution of “Ren” and “Li.”

**Figure 2:**
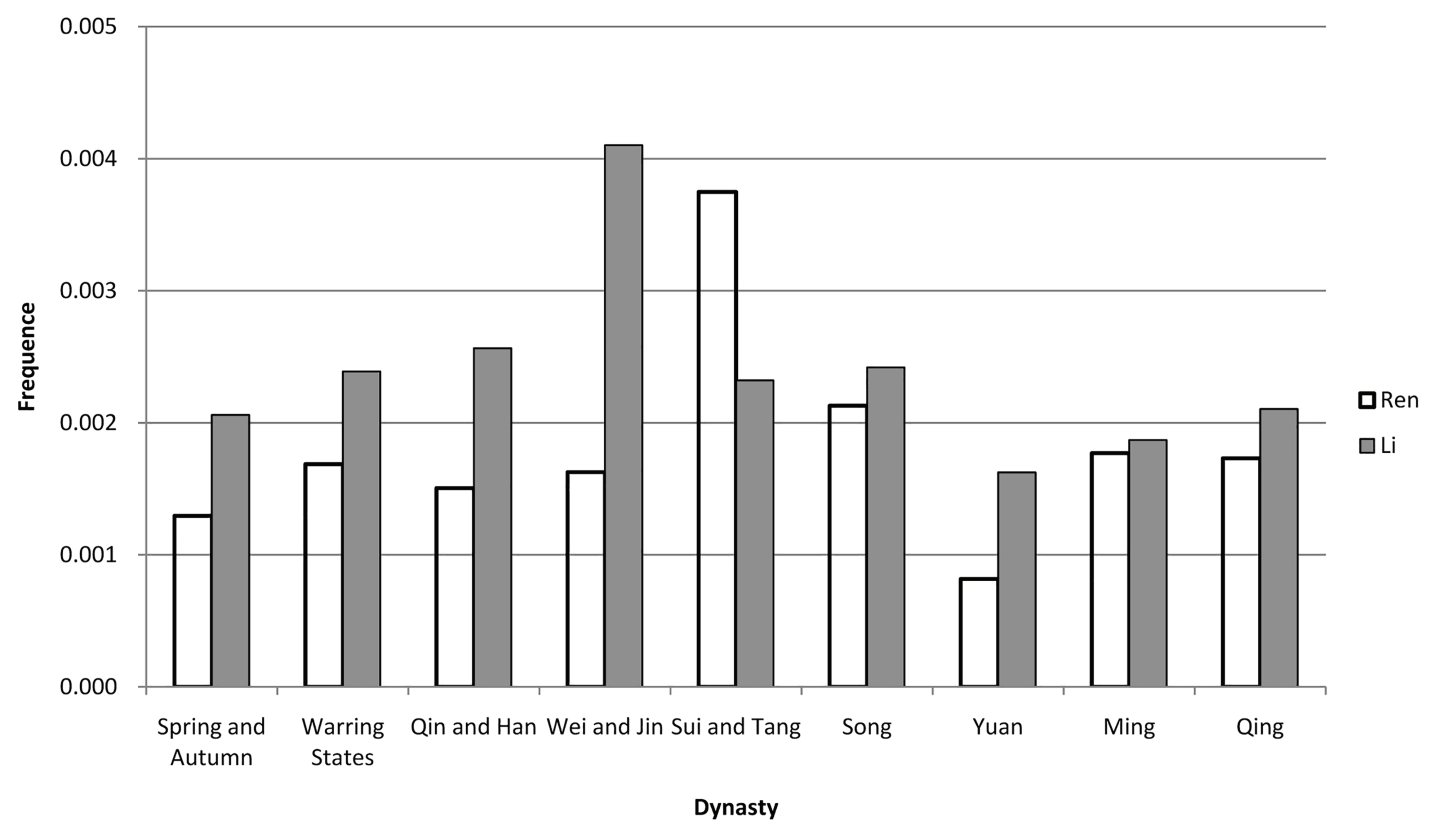
Differences between “Ren” and “Li.”

Second, we divided the number of occurrences of a keyword by a book’s total number of words to calculate the use of frequency of the keyword in that book, after that, we calculated the average frequency of a keyword in all the books of the same dynasty or period (see Table 2). Based on the above calculations, line charts can be generated with the x-axis representing dynasty and the y-axis representing the frequency of a keyword (see Figure 1). In order to further show the differences between “Ren” and “Li,” we use bar chart to display their average frequencies in different dynasties or periods (see Figure 2).

**Table 2:** Average frequency of each keyword in different dynasties and periods.

Dynasty or Period	Average frequency of “Ren”	Average frequency of “Li”
Spring and Autumn (770–476 BC)	0.0013	0.00206
Warring States (475–221 BC)	0.00169	0.00239
Qin and Han (BC 221–220)	0.00151	0.00256
Wei and Jin (220–420)	0.00163	0.0041
Sui and Tang (581–907)	0.00375	0.00232
Song (960–1279)	0.00213	0.00242
Yuan (1279–1368)	0.00082	0.00162
Ming (1368–1636)	0.00177	0.00187
Qing (1636–1912)	0.00173	0.0021

Each number is rounded up to five decimal places; “Dynasty or Period” is listed in chronological order.

**Figure 3:**
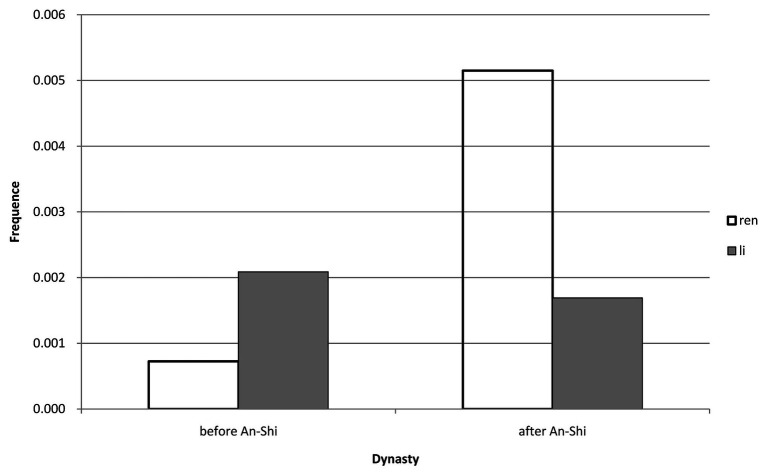
The difference before and after An-Shi disturbances in Tang Dynasty.

Third, considering that the Sui and Tang period (581–907) is the only period during which the frequency of “Ren” is higher than that of “Li” (see Table 2) and that the Classical Prose Movement following the An-Shi Disturbances (755–763) had great impacts on the evolution of Confucianism, we further calculated the average frequencies of “Ren” and “Li” in Tang’s Confucian classics before and after the An-Shi Disturbances to study their differences (see Figure 3).

Fourth, we took the An-Shi Disturbances as a cutting point to conduct a phased analysis of the differences between the average frequency of “Ren” and “Li” in the pre-An-Shi Disturbances era, the post-An-Shi Disturbances era, and the era from the Spring and Autumn period (770–476 BC) to the Qing Dynasty (1636–1912; see Table 3).

**Table 3:** *T*-value between “Ren” and “Li” in different eras.

Era	Quantity of books	Average frequency of “Ren”	Average frequency of “Li”	*T*-value
Pre-An-Shi	32	0.00153	0.00257	−2.44664^∗^
Post-An-Shi	100	0.00199	0.00215	−0.96811
From Spring and Autumn to Qing	132	0.00188	0.00225	−2.27637^∗^

Each number is rounded up to five decimal places.

∗ *p* < 0.05.

## Results

Table 1 shows the number of Confucian works by dynasty or period and the numbers of occurrences of “Ren” and “Li” in them. As shown in the table, the Song (960–1279), Ming (1368–1636) and Qing (1636–1912) dynasties are the top three dynasties in terms of the number of Confucian works, whereas the pre-Song era and the Yuan Dynasty (1279–1368) come with fewer. Accordingly, the numbers of occurrences of “Ren” and “Li” also record high in Confucian works of the Song, Ming and Qing dynasties and low in those of the pre-Song era and the Yuan Dynasty. Notably, the number of occurrences of “Li” is higher than that of “Ren” in Confucian works of all dynasties and periods.

Table 2 shows the average frequency of “Ren” and that of “Li” in different dynasties and periods. As shown in the table, “Ren” reaches its peak in the Sui and Tang Dynasty (581–907; *M* = 0.00375, *SD* = 0.00141) and hits its bottom in the Yuan Dynasty (1279–1368; *M* = 0.00082); “Li” reaches its peak in the Wei and Jin (220–420; *M* = 0.0041, *SD* = 0.00127) and hits its bottom in the Yuan Dynasty (1279–1368; *M* = 0.00162).

As shown in Figures 1, 2, we can see that: (1) from the Spring and Autumn period (770–476 BC) to the Qing Dynasty (1636–1912), both the frequencies of “Ren” and “Li” record a peak and a bottom: “Ren” has its peak in the Sui and Tang period (581–907) while “Li” reaches its climax in the Wei and Jin period (220–420), and both “Ren” and “Li” hit their bottom during the Yuan Dynasty (1279–1368); (2) the frequency of “Li” is higher than that of “Ren” in most of the dynasties and periods, especially during the pre-Sui and Tang era; and (3) the exception takes place in the Sui and Tang period when the frequency of “Ren” becomes higher than that of “Li.”This exception ends after the Sui and Tang period, though the differences between the frequencies of “Ren” and “Li” narrow down.

Figure 3 shows the differences between the frequencies of “Ren” and “Li” in Tang’s Confucian books before and after the An-Shi Disturbances. As we can see, the frequency of “Ren” (*M* = 0.00073) before the Disturbances is lower than that of “Li” (*M* = 0.00209) while the former (*M* = 0.00515) turns higher than the latter (*M* = 0.00169) after the Disturbances.

According to our phased analysis of the average frequency differences between “Ren” and “Li” as shown in Table 3, the average frequency of “Ren” is lower than that of “Li” in the pre-An-Shi Disturbances era (*t* = −2.44664, *p* = 0.02); there is no significant difference between them in post-An-Shi Disturbances era (*t* = −0.96811, *p* = 0.33). Overall, the average frequency of “Ren” is lower than that of “Li” throughout 9 dynasties and periods from the Spring and Autumn period (770–476 BC) to the Qing Dynasty (1636–1912; *t* = −2.27637, *p* = 0.02).

## Discussion

This paper examines a new measure of researching the evolutionary trajectory of “Ren” and “Li” based on the big data of classical Chinese, overcoming the difficulty^2^ of analyzing massive classical Chinese materials that qualitative research cannot well manage. As shown in the results of the data analysis, from the Spring and Autumn period (770–476 BC) to the Qing Dynasty (1636–1912), both the frequencies of “Ren” and “Li” record a peak and a bottom: “Ren” has its peak in the Sui and Tang period (581–907), while “Li” reaches its climax in the Wei and Jin period (220–420), and both “Ren” and “Li” hit their bottom during the Yuan Dynasty (1279–1368; see Figure 1).

The first notable period is the Wei and Jin period (220–420), during which the frequency of “Li” surges (see Figure 1). Some scholars believe that it is “Li” that underpins the society dominated by powerful families in the Wei and Jin period, since “Li” defines the differences between social classes (Feng, 2018, p. 2). According to Mancang Liang, Vice Chairman of the Historical Association of Wei-Jin and Southern and Northern Dynasty of China, the national implementation of the five systems of “Li” started in the Wei and Jin period, marking a milestone for the social status change of “Li” (Liang, 2001, p. 3, 22–24). These explanations may echo the peak of the frequency of “Li” during the Wei and Jin period.

The second conspicuous period is the Sui and Tang Dynasty period (581–907), during which the frequency of “Ren” climbs and that of “Li” plummets (see Figures 1, 2). Specifically, during the Tang Dynasty, the frequency of “Ren” is lower than that of “Li” before the An-Shi Disturbances and the former becomes higher than the latter after the Disturbances (see Figure 3). It is generally recognized that the An-Shi Disturbances marks a turning point for the Tang Dynasty, and the Classical Prose Movement following the Disturbances has significant impacts on the Chinese culture (Chen, 2019, p. 8). Some scholars believe that the Classical Prose Movement serves as an important driver for the revival of Confucianism (Tong, 2009, p. 1). On the one hand, Yu Han and Zongyuan Liu, the main representatives of the Classical Prose Movement, expressed dissatisfaction with “Li” at that time. They advocated inheriting the Pre-Qin Confucianism and denied the official Confucianism adopted since the Han Dynasty (BC 206–220; Feng, 2018, p. 1). Meanwhile, powerful families that had dominated the society began to decline after the An-Shi Disturbances; as a result, scholar-officials from common families began to resist the ideological “Li” (Feng, 2018, p. 2). On the other hand, the pre-Qin Confucianism advocated by Yu Han et al. is clearly different with the Han Confucianism as follows: the former stresses the peering relationship between the rights and obligations of a monarch and his subjects while the latter tends to safeguard the hierarchical ruling and socialorder (Chen and Ma, 2018, p. 3); the former takes “Ren” as its core, advocating benevolent governance, while the latter promotes the syncretism of “Li” and law (Han and Shan, 2019, p. 1). We can then believe that the Classical Prose Movement may be the reason why the frequency of “Ren” turns higher than that of “Li” during the Sui and Tang period.

The third noteworthy period is the Yuan Dynasty (1279–1368) in which both the frequencies of “Ren” and “Li” drop to a low point. The Yuan Dynasty is the first dynasty in the history of China that is ruled by an ethnic group outside the Han people. Since the ruling times of Kublai, the first emperor of Yuan, who walked far away from Confucian scholars and even suppressed them, this dynasty estranged itself from Confucianism and finally renounced it (Shen, 2006, p. 1). Even though emperors after Kublai valued Confucianism at some point, the Yuan Dynasty, as a whole, put the military above civilian rule and even canceled the imperial examinations repeatedly. Moreover, the dynasty implemented ethnic discriminatory policies, further downgrading the status of Confucian scholars. All these impeded the development of Confucianism during this period, there by resulting in the low points of both “Ren” and “Li.”

By calculating the changing trends of use of frequency of “Ren” and “Li” over time, we have observed several important nodes in the development of Confucianism and provides an overall perspective for understanding the evolution of “Ren” and “Li.” However, it is worth further asking, which one of “Ren” and “Li” is more frequently referred to in classic Confucian works?

To answer this question, we have made a hypothesis earlier. According to our calculation, the average frequency of “Ren” is lower than that of “Li” in the pre-An-Shi Disturbances era; there is no significant difference between them in the post-An-Shi Disturbances era. Overall, the average frequency of “Ren” is lower than that of “Li” throughout nine dynasties and periods from the Spring and Autumn period (770–476 BC) to the Qing Dynasty (1636–1912; see Table 3). We can then deduce the hypothesis stands, that is, “Li” is more frequently referred to in Confucian classics than “Ren,” especially in those of the pre-An-Shi Disturbances era, and the An-Shi Disturbances may mark an important turning point for the frequencies of “Ren” and “Li” in classic Confucian works. Based on the differences in the frequencies of “Ren” and “Li” in Confucian classics, we believe that Confucian works seem to place more emphasis on institutions and behavioral norms, reflecting that its focus is more on pragmatic application than on theoretical thinking.

There are several additional limitations in our study that merits attention. The 132 books selected by this paper from *SikuQuanshu* do not cover all Confucian works and subjective judgment may play a part in the selection. It might also be noted that the number of Confucian classics is relatively small during the Wei and Jin, Sui and Tang, and Yuan dynasties, which may affect the results of keyword frequencies to certain extent; in addition, the most important writers of an epoch are usually ahead of their times, some Confucian works does not necessarily represent the general opinions of his epoch, which can be a challenge for this paper to explain the results of frequencies of keywords. Moreover, the same Chinese character may carry different meanings under different contexts, and the meanings and connotations of “Ren” and “Li” have been changing slightly over time with the development of Confucianism, which may affect this research to some extent. In short, quantitative research is not set against qualitative research; rather, it provides a new perspective and supplementary information for previous research. There is great potential to apply big data to future studies of the evolution of Confucian culture. For instance, big data can be used to analyze the development of the “three principles and five virtues” and compare the cultural differences of “loyalty” and “filial piety” between the pre-Neo-Confucianism period and the Neo-Confucianism period. Such innovative approaches based on big data are worthy of further studies.

## Conclusion

This paper analyzed the evolution of “Ren” and “Li” in the Confucian culture based on big data. In the past over 2,000 years, both the frequencies of “Ren” and “Li” record a peak and a bottom: “Ren” has its peak in the Sui and Tang period (581–907), while “Li” reaches its climax in the Wei and Jin period (220–420), and both “Ren” and “Li” hit their bottom during the Yuan Dynasty (1279–1368). In general, “Li” is more frequently referred to in classic Confucian works than “Ren,” especially in those of the pre-Sui and Tang era. The An-Shi Disturbances in the Tang Dynasty may mark an important turning point for the frequencies of “Ren” and “Li” in classic Confucian works.

## Data Availability Statement

The original contributions presented in the study are included in the article/Supplementary Material, further inquiries can be directed to the corresponding author.

## Author Contributions

TZ designed this research and provided key guidance throughout the writing of this paper. BH wrote the manuscript. MF and FX provided the CC-LIWC program for keyword extraction and contributed to the collection of primary data. All authors contributed to the article and approved the submitted version.

### Conflict of Interest

The authors declare that the research was conducted in the absence of any commercial or financial relationships that could be construed as a potential conflict of interest.

## Footnotes

^1^According to the definition of “classics” given above, this paper views all the 113 Confucian works from the Zi category of *SikuQuanshu* as Confucian classics; it also selects 19 works mainly from the Jing and Ji categories of *SikuQuanshu*. Undeniably, subjective judgment (on the importance of a work, for instance) may play a part in the selection.

^2^Quantitative research is good at processing a large volume of materials in a relatively short time, but researchers still rely on qualitative methods to explain the results found through quantitative research.
